# Infective endocarditis: it takes a team

**DOI:** 10.1093/eurheartj/ehaf219

**Published:** 2025-04-11

**Authors:** Lawrence Lau, Larry Baddour, Núria Fernández Hidalgo, Thomas D Brothers, William K F Kong, Michael A Borger, Xavier Duval, Christophe Tribouilloy, Jean-Francois Obadia, Mehrdad Golian, Vicente F Corrales-Medina, Francois Auclair, Mikael Mazighi, Kwan Leung Chan, Bernard Prendergast, Gilbert Habib, Fraser D Rubens, David Messika-Zeitoun

**Affiliations:** Division of Cardiology, University of Ottawa Heart Institute, 40 Ruskin Street, K1Y 4W7 Ottawa, Ontario, Canada; Departments of Medicine and Cardiovascular Medicine, Division of Public Health, Infectious Diseases and Occupational Medicine, Mayo Clinic College of Medicine and Science, Mayo Clinic, Rochester, MN, USA; Servei de Malalties Infeccioses, Hospital Universitari Vall d’Hebron, Barcelona, Vall d’Hebron Institut de Recerca (VHIR), Vall d’Hebron Barcelona Campus Hospitalari, Passeig de la Vall d’Hebron 119-129, 08035 Barcelona, Spain; Departament de Medicina, Universitat Autònoma de Barcelona, Barcelona, Spain; CIBERINFEC, ISCIII-CIBER de Enfermedades Infecciosas, Instituto de Salud Carlos III, Madrid, Spain; Division of General Internal Medicine, Dalhousie University, Halifax, Nova Scotia, Canada; Addiction Medicine Consult Service, Queen Elizabeth II Health Sciences Centre, Nova Scotia Health, Halifax, Nova Scotia, Canada; Department of Cardiology, National University Heart Centre, National University Health System, Singapore, Singapore; Leipzig Heart Center, University of Leipzig, Leipzig, Germany; Université Paris Cité and Université Sorbonne Paris Nord, Inserm 1137, IAME, F-75018 Paris, France; Inserm CIC 1425, Centre d’investigation Clinique, AP-HP, Hôpital Bichat, F-75018 Paris, France; Department of Cardiology, Amiens University Hospital, Amiens, France; Chirurgie Cardio-Vasculaire et Transplantation Cardiaque, Hôpital Cardiovasculaire Louis Pradel, 28, Ave. Doyen Lépine, 69677 Bron CEDEX, France; Division of Cardiology, University of Ottawa Heart Institute, 40 Ruskin Street, K1Y 4W7 Ottawa, Ontario, Canada; Division of Infectious Disease, The Ottawa Hospital and University of Ottawa, Ottawa, Ontario, Canada; Division of Infectious Disease, The Ottawa Hospital and University of Ottawa, Ottawa, Ontario, Canada; Université Paris Cité, Institut Universitaire de France, Département de Neurologie, Hôpital Lariboisière, et Service de Neuroradiologie Inteventionnelle, Hôpital Fondation Rothschild, FHU NeuroVasc, INSERM UMRS 1144, Paris, France; Division of Cardiology, University of Ottawa Heart Institute, 40 Ruskin Street, K1Y 4W7 Ottawa, Ontario, Canada; Heart Vascular and Thoracic Institute, Cleveland Clinic London, London, UK; APHM, La Timone Hospital, Cardiology Department, Aix Marseille Université, Marseille, France; Division of Cardiac Surgery, University of Ottawa Heart Institute, Ottawa, Ontario, Canada; Division of Cardiology, University of Ottawa Heart Institute, 40 Ruskin Street, K1Y 4W7 Ottawa, Ontario, Canada

**Keywords:** Infective endocarditis, Team, Multidisciplinary

## Abstract

Infective endocarditis (IE) is a relatively rare but life-threatening systemic infection, which remains associated with high morbidity and mortality. The epidemiology of IE has shifted to involve an increasing numbers of older patients with both cardiovascular and other types of prosthetic devices, multiple comorbid conditions often requiring invasive procedures, increasingly virulent pathogens, in particular *Staphylococcus aureus*, or that can harbour anti-microbial resistance, and an escalation of injection drug use in many areas of the world. In parallel, advancements in diagnostic and therapeutic options have led to complex strategies in patients’ management. Despite these epidemiologic shifts, clinical trials have been rare and most of the evidence guiding IE management derives from expert consensus or analysis of large registries. Because of this, a multi-disciplinary IE team-based approach has been recommended as the standard of care. The aim of this review is to explore the rationale for a multi-disciplinary team-based approach to the management of IE. This approach has proved to be potentially beneficial based on multiple investigations that have evaluated patient outcomes. In addition, implementation strategies, feasibility and options of the team approach have also been highlighted.

## Graphical Abstract

**Graphical Abstract:**
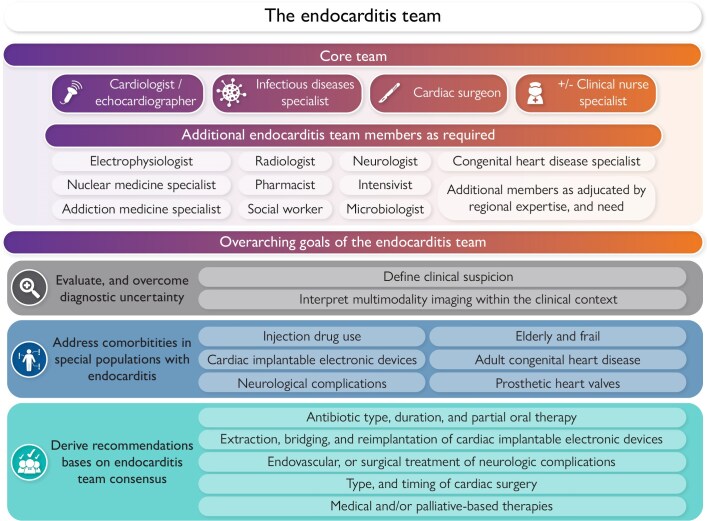

## Introduction

Infective endocarditis (IE) was first described in the 18th century in an era when autopsy was the primary method of diagnosis.^1^ Although the diagnosis and treatment of this disease has since markedly improved, IE remains associated with significant mortality and morbidity.^2^ The current epidemiology of IE has shifted to affect patients who have significant comorbidities, are frail, inject drugs, or who have underlying structural heart disease and cardiovascular devices, often in the setting of healthcare-related infections. Moreover, the prevalence of IE due to *Staphylococcus aureus*, a virulent pathogen associated with worse outcomes, has increased.

The multi-disciplinary team-based approach has become the standard of care for patients with complex cardiovascular disease, including IE, which frequently affects multiple organ systems with protean clinical manifestations. Integration of diverse expertise from multiple areas of care ensures that patients with IE receive coordinated, standardized, and comprehensive care especially, particularly given the relatively rare nature of the disease. Owing to limited evidence and wide variability in disease presentation, management decisions are often based upon expert consensus.^3–5^ Endocarditis teams (ET) provide a platform for expert case-by-case discussions, with increased recognition of their importance over the last decade. Several ET models have demonstrated improved outcomes, yet their diversity highlights that there is no one-size-fits-all approach. The aims of this paper are to review the rationale for a multi-disciplinary team-based approach to the diagnosis and management of IE, to highlight steps and components that are essential to successful ET implementation and to suggest methods to increase their adoption.

## Regional variability of epidemiology in infective endocarditis

The epidemiology of IE demonstrates significant regional variability, reflecting several factors including access to advanced medical care, geographical patterns of injection drug use, and areas where rheumatic fever continues to have a significant public health impact.^6^ Although not unique to IE, socioeconomic factors also significantly impact on the availability of therapeutic options and outcomes.^7^ In high-income countries, advances in medical care have changed the demographics of those affected by IE, with an increasing proportion of older patients being affected with IE over time.^8^ Advanced age, chronic kidney disease requiring haemodialysis, malignancy, and rheumatologic disease are associated with an increased risk of IE.^9^ Patients with these risk factors often have more frequent health care contact and may require long-term intravenous access, increasing the risk of blood steam infections.^10^ Changes in the epidemiology of substance use disorders (SUDs) and social determinants of health^11^ have contributed to a rising incidence of IE in people who inject drugs in Europe,^12^ Australia,^13^ and North America.^14^ Although these patients have lower mortality compared with non-IDU-associated IE patients, they have a higher rate of relapse and recurrence.^15–17^ Poor outcomes in this subset are often the result of underappreciation of psychosocial complexities, inadequate treatment of the underlying SUD, and sub-optimal provision of surgical therapy.^18,19^ Rheumatic heart disease remains an important risk factor for endocarditis in low- and middle-income countries.^20^ The growing use of implanted cardiovascular hardware, including transcatheter aortic valve implantation (TAVI) and cardiac implantable electronic devices (CIED) has also contributed to a growing prevalence of IE and poses unique diagnostic and therapeutic challenges.^21–23^ The evolving microbiological profile of IE reflects these demographic changes. Contemporary registry data show that *S. aureus* and *Enterococcus spp.* have now overtaken *viridans* group streptococci as the most frequent IE causative organisms, and are particularly prevalent in prosthetic valve IE and TAVI-associated IE.^8,10,21^ This trend is especially notable in high-income countries, but is also observed in low- to middle-income countries.^20^

## Rationale for a dedicated endocarditis heart team

Although the global incidence of IE has increased over the past 30 years,^24^ IE remains a relatively rare condition, affecting about 15 per 100 000 of the population.^25^ As a result, large international registries, such as the International Cohort of Endocarditis and EURO-ENDO cohort registries, are required to meaningfully study this disease.^6,8^ Recognition of IE is challenging and diagnosis often delayed from symptom onset.^26^ On the other hand, patients may first present with symptoms related to severe or catastrophic cardiac or extra-cardiac complications.^27,28^ While echocardiography can readily diagnose native valve IE, it can only be performed if IE is suspected. Conversely, prosthetic valve IE can be difficult to confirm with imaging even when it is suspected. Decision-making is challenging, but collaboration can overcome the steep learning curve.

**Figure 1:**
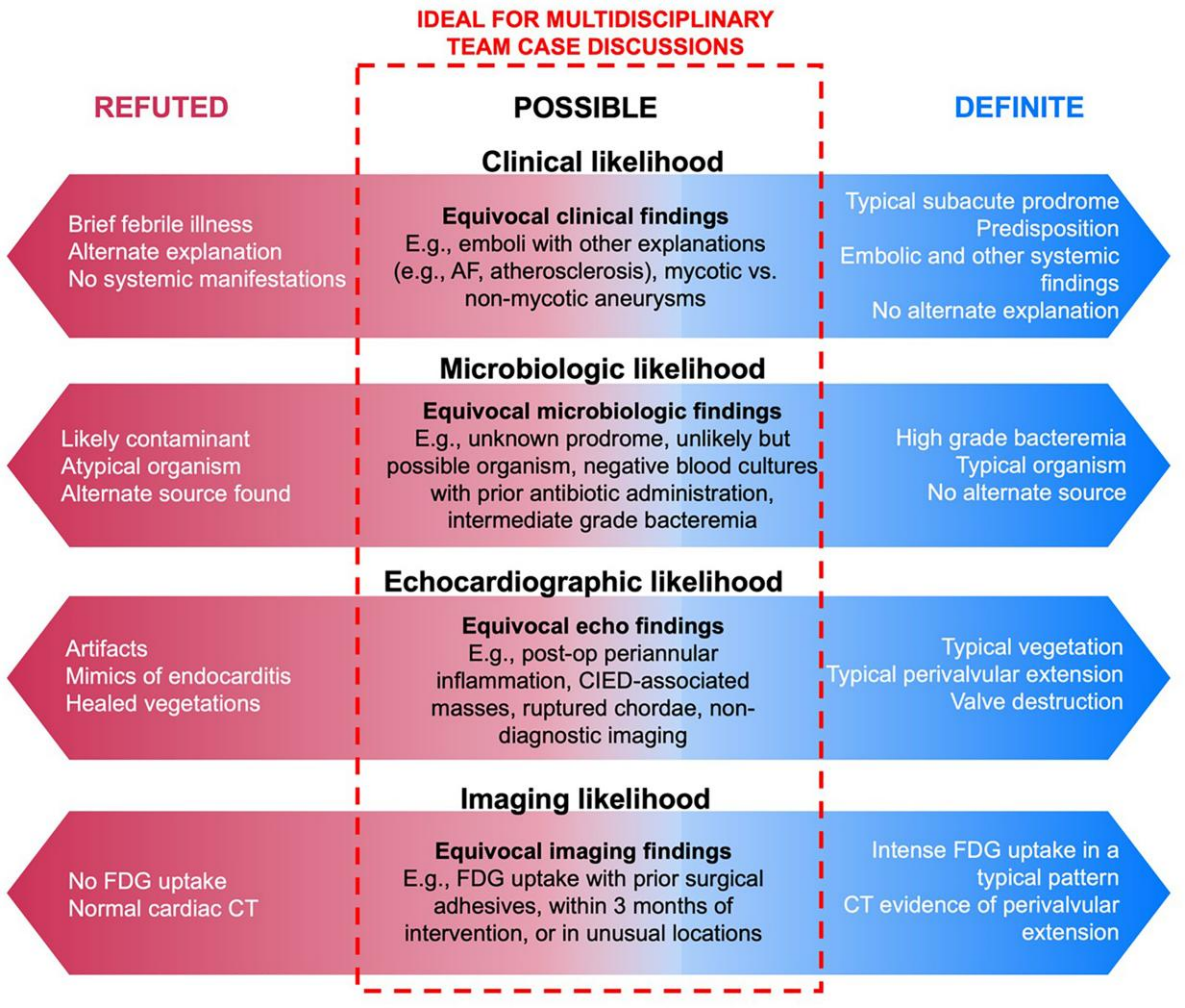
Probabilistic classification of data points used in the diagnosis of infective endocarditis. The dashed box highlights situations in which the endocarditis team provides the highest additional diagnostic value

The modified Duke diagnostic criteria,^29^ the 2015 ESC criteria,^30^ and the 2023 Duke-ISCVID criteria^31^ all categorize the clinical likelihood of IE as definite, possible, or refuted. This probabilistic classification reflects the inherent imperfection of any one diagnostic test or clinical finding. Rather, the final diagnosis represents the combined probability taking all individual certainties and uncertainties into account (*Figure 1*). Nevertheless, diagnosis remains only possible in a significant proportion of patients, which can vary between 11% and 35% according to series.^32–35^ Review by a ET is useful to reach a consensus diagnosis when the diagnostic probability is intermediate or possible (*Figure 1*, dotted box). Updated diagnostic criteria include 18-F fluorodeoxyglucose positron emission tomography (FDG PET), leucocyte scintigraphy, and computed tomography (CT) as additional means to reach a definite diagnosis in complex or prosthetic valve infections.^5,22,31^ However, access to these advanced imaging modalities is often limited to larger referral institutions with available resources and expertise and their interpretation can be subjective. Using FDG PET as an example, interpretation requires consideration of both intensity and pattern of FDG uptake to infer a diagnosis of IE. Clinical history and sound knowledge of technical factors are also important to explain false positive findings and to rationalize a negative result when the clinical suspicion is high.^36,37^ The contextualized, multi-disciplinary decision-making by the ET makes the consensus diagnosis reached by the ET the gold standard against which accepted diagnostic criteria or new diagnostic tools are validated against.^35^ It can also be used to determine the significance of clinical findings such as rapid or delayed defervescence.^38^

Adherence to established IE guidelines is often sub-optimal reflecting both the inherent variability in IE disease presentation and the low level of evidence supporting current guideline recommendations. In contrast to many other cardiovascular conditions, evidence in IE is largely limited to observational studies and expert consensus. Few large randomized trials have been performed in patients with IE with notable exceptions: the EASE trial, which supports early surgery in patients with an established indication to prevent systemic embolism,^39^ and the POET trial, which suggests that partial oral antibiotic therapy is non-inferior to full-course intravenous therapy in certain indications.^40^ Ongoing clinical trials include RODEO-1 and RODEO-2, which will expand on the role of oral switch during antibiotic treatment,^41^ and POET II, which will investigate the safety of shortened antibiotic therapy duration.^42^

Despite evidence showing that routine consultations with the cardiology and infectious disease (ID) services improve survival, they are sometimes omitted.^43^ Echocardiography, especially transoesophageal echocardiography (TOE), is underused, particularly in elderly patients.^44^ Adherence to guideline recommendations on antibiotic therapy is also sub-optimal,^45^ with significant variability in antibiotic use even amongst ID specialists.^46^

Although there is significant regional and centre-specific variability, only about 25%–50% of patients are managed surgically.^47–49^ Despite evidence of the benefit of surgery in reducing embolic events and clear guideline recommendations, surgical indications are commonly under-recognized.^8,50–53^ There may also be a bias of being considered either too stable to require surgery or too clinically ill to survive surgery. Often, false perceptions of excess frailty or comorbidity hinder surgery, especially in elderly patients.^54^ Stigma or misunderstanding may also contribute to lower rates of surgery offered to people who inject drugs.^19,55,56^

Microbiologic diagnosis, antibiotic selection and duration, management of systemic complications,^57^ nature of follow-up based on risk of relapse or recurrence,^15^ and use of long-term antibiotic therapy when surgery is indicated but not performed^58^ are among the critical elements of non-operative management that are based on limited but evolving data. Newer anti-microbial and other non-surgical interventions are increasingly used. Dalbavancin has shown promise as a consolidative antibiotic in IE,^59^ and may reduce length of hospital stays and obviate the need for indwelling intravenous catheters.^60^ Percutaneous vacuum-assisted aspiration of vegetation or thrombi may be considered in selected patients as an adjunctive or alternative treatment option where the technology and expertise are available although the level of evidence remains low.^61^ The ET may be a useful platform for randomized controlled studies to better define the indications, risks, and benefits of these off-label and/or new therapies. It must be acknowledged that guidelines should be adapted to align with geographic or institutional practices where necessary, understanding that trial data may not accurately reflect the real-world practices.^45,62,63^ In this context, ET are well-positioned to implement guideline recommendations within an individualized framework suited to each institution within its specific geographical setting.

## Evidence supporting endocarditis teams

The model of multi-disciplinary teams was first integrated into oncology as standard practice^64^ and has since spread across many fields, including cardiology. Heart teams have established roles in the management of patients with complex coronary artery disease, heart failure and transplantation, and valvular heart disease.^65^ International cardiovascular/IDs societies have ascribed a strong level of recommendation for multi-disciplinary teams in the management of IE.^3,5^ The primary goal of the ET is to expedite the diagnosis and treatment of IE patients. Longitudinal follow-up by the ET is often required to re-evaluate the effectiveness of the approach. ET adoption, while growing, remains sub-optimal.^3,30^ Outcomes from several ET across Europe and North America have been published and their structure, patient volumes, and outcomes are summarized in *Table 1*. Most ET consist of a core group of cardiologists, cardiac surgeons, and IDs specialists. More recently, specialists in nuclear medicine, neurology, radiology, addiction medicine (AM), geriatric medicine, and pharmacy have been included,^88,89^ which reflects the increasing use of advanced cardiac imaging modalities, complexities of managing extra-cardiac complications, new treatment paradigms, and regional variability in epidemiology. Most teams convene on a weekly basis, while a few incorporate *ad hoc* discussions for urgent cases. Cases are primarily identified through consultant specialists involved in patient care. A few teams served as regional referral centres and reviewed cases admitted to primary care hospitals.

**Table 1:** Summary of published endocarditis team data

Author and year	Country	Dates of ET	Format	Before (*n*)	After (*n*)	Use of imaging	IE diagnosis and specific inclusion criteria	Use of cardiac surgery (after ET vs before ET)	Mortality (after ET vs before ET)	Additional reported outcomes
Before and after observational studies (ordered by descending year of publication)										
Fourré *et al.*^66^	Switzerland	2018–2022	Weekly	187	318	91% TTE, 81% TOE, 28% PET, 7% CT		78% vs 66%, *P* = .038	No differences in 30-day mortality (13% vs 15%, *P* = .60) or 1-year mortality (26% vs 29%, *P* = .53)	Significant increase in PET use (28% vs 14%, *P* < .001), operation when surgery is indicated (78% vs 66%, *P* = .038), and shortened antibiotic duration (55% vs 85%, *P* < .001 with more than 4 weeks antibiotic duration)
Crosby *et al*.^67^	United States	2018–2020	NR	21	31	NR	100% definite; included patients with at least 1 surgical indication	55% vs 29%, *P* = .061	No difference in in-hospital mortality (16% vs 29%, *P* = .28)	Non-significant trend towards increased surgery when indicated (55% vs 29%, *P* = .06)
van den Heuvel *et al*.^33^	Netherlands	2019–2020	Weekly	45	45	99% echo, 51% PET	76% definite, 22% possible	71% vs 69%, *P* = .82	No difference in 6-month mortality (11% vs 13%, *P* = .75)	No difference in diagnosis, surgery, time to surgery, in-hospital complications.
Elad *et al*.^68^	Israel	2016–2019	*Ad hoc* only	92	128	89% TOE, 41% CT, 36% PET	100% definite	35% vs 32%, *P* = .71	Reduced short- and long-term mortality. 8.5% vs 17.4% (*P* = .048) at 30 days; 28% vs 37% (*P* = .121) at 1 year, 35% vs 48% (*P* = .064) at 3 years.	Reduced time to investigations; higher rates of surgery and CIED extraction. Higher readmission rates in ET group
El-Dalati *et al*.^69^	United States	2018–2019	Weekly	68	56	NR	100% definite; included patients with at least 1 surgical indication	31% vs 28%, *P* = .12	Reduced in hospital mortality (7.1% vs 29.4%, *P* < .0001)	Reduced LOS; change in antibiotics in 63%; non-significant change in medical to surgical therapy in 20%.
Pecoraro *et al*.^34^	South Africa	2019–2021	NR	75	76	NR	74% definite	51% after ET; before ET NR	Non-significant trend towards lower in-hospital mortality in culture-negative IE (14% vs 23%, *P* = .35). No difference in in-hospital mortality in culture-positive IE (13% vs 18%, *P* = .65).	Reduced incidence of culture-negative IE with no organism detected (14% vs 57%, *P* < .01)
Van Camp *et al*.^70^	Belgium	2017–2019	Weekly	68	92	NR	100% definite	51% (includes both before ET and after ET)	No difference in in-hospital, 30-day, or 1-year mortality. Trend towards reduced adjusted mortality at 1 year (26.5% vs 41.2%, *P* = .0699).	Reduced LOS.
Diab *et al*.^71^	Germany	2011–2018	*Ad hoc* (within 6 h)	732	389	NR	100% definite (all had cardiac surgery)	All had cardiac surgery	Reduced in-hospital mortality (18% vs 32%, *P* < .001) and 1 year mortality (29% vs 44%, *P* < .001).	Reduced incidence of post-op stroke and haemodialysis.
Molnar *et al*.^72^	Romania	2015–2018	NR	214	92	NR	100% definite (all had cardiac surgery)	All had cardiac surgery	No difference in early post-op mortality after emergency or urgent surgery (17% vs 32%, *P* = .21)	Non-significant increase in emergency or urgent surgery (25% vs 19%, *P* = .25)
Sadeghpour *et al*.^73^	Iran	2016–2019	Weekly or biweekly	445	200	NR	All definite or possible	55% vs 56%, *P* = .7	Reduced in-hospital mortality (13% vs 18%, *P* = .05)	Higher rate of positive blood culture (76% vs 43%, *P* = .001), reduced incidence of cerebral emboli (18% vs 39%, *P* < .01), heart failure (36% vs 53%, *P* < .01), new abscess formation (7% vs 17%, *P* < .01), and vascular mycotic aneurysm (1% vs 2%, *P* = .01)
Ruch *et al*.^74^	France	2017	Weekly	316	75	NR	100% definite Excluded VAD patients.	45% vs 48%, *P* = .70	Non-significant trend in reduced in-hospital mortality (14.7% vs 20.3%, *P* = .27) and 1-year mortality (23.4% vs 16%, *P* = .70)	Reduced time from definite diagnosis to surgery; reduced LOS.
Tan *et al*.^75^	Canada	2015–2017	*Ad hoc* (electronic or in person)	97	80	NR	100% definite Excluded patients who declined surgery.	35% vs 22%, *P* = .06	Similar in hospital (22.5% vs 13.4%, *P* = .16) and 90-day mortality (26.3% vs 17.5%, *P* = .20)	Non-significant reduction in complications, HF, critical care admission.
Kaura *et al*.^76^	United Kingdom	2012–2015	Immediate discussion, followed by weekly meetings	101	95	NR	100% definite	66% vs 73%, *P* = .55 for management strategy	Improved 1 year survival in medically managed patients (66.7% vs 42.9%, *P* = .03) but not surgically managed.	Reduced time to TOETOE, time to starting IE-specific antibiotics, and time to surgery; reduced LOS.
Carrasco-Chinchilla *et al*.^77^	Spain	2008–2011	*Ad hoc*	155	72	100% TTE, 74% TOE	100% definite Included left-sided IE only.	56% vs 46%, *P* = .20	Lower operative mortality (13.5% vs 37.7%, *P* = .01) and mortality undergoing early surgery (14.3% vs 33.3%, *P* = .06). Lower in-hospital mortality/mortality 1 month after discharge (16.7% vs 36.1%, *P* = .003).	Increased uptake of cardiac surgery and early surgery; reduced incidence of septic shock; trend towards reduced neurologic complications and heart failure.
Chirillo *et al*.^78^	Italy	2003–2009	*Ad hoc* (within 12 h)	190	292	100% TTE and TOE	100% definite Included native valve IE only.	43% vs 31%, *P* = .06	Lower in-hospital mortality (13% vs 28%, *P* = .02) due to less septic shock and lower operative mortality. Lower 3-year mortality (16% vs 34%, *P* = .0007)	
Botelho-Nevers *et al*.^79^	France	2002–2006	Same day consultation with ID and cardiology	173	160	100% TTE and TOE	100% definite	54% vs 54%, *P* = .54	Lower in-hospital (4.4% vs 12.7%, *P* = .007) and 1-year mortality (8.2% vs 18.5%, *P* = .008).	Reduced incidence of multi-organ failure and renal failure. Improved antibiotic and surgery compliance.
Single-cohort observational studies										
Avogadri *et al*.^35^	Sweden	2017–2022	NR		618	82% TOE, 8.5% CT, 13% PET	51% definite, 35% possible, 14% rejected	28%	11% 30-day mortality	Using ET decision to treat as IE as reference standard, Duke-ISCVID criteria was slightly more sensitive compared with modified Duke or ESC 2015 criteria.
Wahadat *et al*.^32^	Netherlands	2016–2020	Biweekly and *ad hoc*		321	47% had PET, 26% had CT	15% rejected, 11% possible, 75% definite	NR	23% (definite), 29% (possible) at 23 months.	Change in diagnosis in 17% of total group (most were possible to rejected); change in management: 42% changed antibiotics, 5% went from conservative to surgery.
Van Hemelrijcka *et al*.^80^	Switzerland	2016–2020	Weekly		595	NR	58% IE, 22% rejected, 12% CIED-IE, 8% vascular graft infection	40%	9.6% 30-day mortality, 14% 1-year mortality	
Pecoraro *et al*.^81^	South Africa	2019–2021	Weekly		72	NR	72% definite, 28% possible	58%	18% in hospital, 26% 6-month; lower than previously reported 6-month mortality at same institution (36%)	Higher rates of MV repair (vs replacement); reduced incidence of culture negative IE (recognized Bartonella as an important cause).
Vyas *et al*.^82^	United States	2018–2020	Monthly and *ad hoc*		57	NR	All definite or possible, all self-reported injection drug use	25%	7% in-hospital mortality	65% discharged on medication for opioid use disorder. 18% patient-directed discharge, 42% readmission within 90 days of discharge.
Hartley *et al*.^83^	United Kingdom	2020 (Mar to Jul)	Weekly		38	87% TTE, 16% TOE, 5% MRI, 0% PET	29% definite, 3% possible, 68% refuted	33%	17% 30-day mortality, 25% 1-year mortality	ET could be used to avoid routine TTE/TOE during the Covid-19 lockdown
Camou *et al*.^84^	France	2013–2017	Weekly		704	88% TOE, 24% PET	70% definite or possible, 30% refuted	49%	12% in-hospital mortality	Higher mortality in elderly patients, absence of surgical treatment, initial heart failure, and *S. aureus*.
Barbieri *et al*.^85^	Italy	2009–2013	Daily review		323	13% TOE	8% positive for IE, 92% IE excluded (echo diagnosis)	73%	19% mortality at mean follow-up of 2.3 ± 1.4 years	Incidence of clinical events was similar between IE and non-IE groups as adjudicated by the 2009 ESC echo diagnostic strategy.
Bain *et al*.^86^	United Kingdom	1976–1984	*Ad hoc*		62	NR	Clinical diagnosis	16%	8% 1-year mortality (of patients managed by ET); 24% mortality in total IE during this period (including diagnoses made post-mortem)	

NR, rot reported; LOS, length of hospital stay; CIED, cardiac implantable electronic device; VAD, ventricular assist device; TTE, transthoracic echocardiography; TOE, transoesophageal echocardiography; PET, positron emission tomography.

Search strategy. We searched Medline (from inception to 15 October 2024) and Embase (from inception to 15 October 2024) with the assistance of a medical research librarian. We focused on Medical Subject Headings and key words related to ‘endocarditis’, ‘multi-disciplinary’, and ‘team’. The search was informed by previously conducted systematic searches.^87^ We included both observational before and after studies and cohort studies published in English that reported mortality as an outcome. Conference abstracts and case reports were excluded.

A recent systematic review and meta-analysis has suggested an outcome benefit of ET.^87^ Despite wide variability in team structure and population of IE patients included within individual analyses, data generally suggest that ET implementation is associated with lower mortality and reduced length of hospital stay (*Table 1*). The increased uptake of guideline-based diagnostic and treatment recommendations, including cardiac imaging, microbiologic techniques, and surgery, has been, hypothesized to account for improved outcomes.^66–69,75,77,78,81^ Critically, these data are observational and as such should be interpreted with caution. The use of an imperfect historical comparator, exclusion of patients who were not reviewed by the ET for early death or palliation, and inclusion of patients who did not have definite IE all impact the validity of reported outcomes.

Some potential benefits of an ET are subjective and harder to quantify. Without the ET, the attending physician is left to independently develop a patient management strategy that may not be optimal due to lack of IE expertise. In our experience, the reassurance and guidance provided by the ET are highly valued. In addition, an ET may be a source of education both for patients and clinicians as well as quality improvement initiatives. For example, through the implementation of a systematic protocol for IE diagnosis, the ET described by Pecoraro *et al*. identified *Bartonella* species as an important, previously under-recognized cause of culture-negative IE at their institution.^81^ Hartley *et al*. described a ET-based approach of using echocardiography selectively rather than routinely during the early phase of the Covid-19 pandemic.^83^ The clinical expertise of a regional referral centre for IE may be disseminated through a specialized, protocolized programme as described by Diab *et al*.^71^ The combination of ET and a prospective registry of IE patients has been associated with improved outcomes.^70^

## Practical considerations for the endocarditis heart team

### Organization and logistics

The core team must designate a cardiologist, ideally with expertise in echocardiography, an IDs specialist, and a cardiovascular surgeon who are committed to regular attendance. Additional specialized expertise may also be identified to meet on a case-by-case basis. The designation of a dedicated co-ordinator for collating referrals, case presentation, and communicating recommendations to the treating team is essential. The role of the ET is to provide non-binding recommendations that need to be understood and shared by the treating team and the treating physician is crucial partner of the ET. He possesses the most comprehensive understanding of the patient’s background, clinical condition, including frailty, and care goals, and ensures that therapeutic plans align accordingly. A direct contact by one or more ET members is also highly valuable.

Methods of patient referral to the ET vary considerably among centres and reflect regional infrastructure and institutional practices. Botelho-Nevers *et al*. describe a medical-surgical protocol in which same-day consultation with cardiology and IDs is routinely carried out on all patients with suspected IE^79^ while Chirillo *et al*. hold *ad hoc* meetings on all referred patients within 12 h of admission or diagnosis.^78^ Van Camp *et al*. identify cases upon admission to the cardiac care unit,^70^ and Diab *et al*. review cases referred by IDs,^71^ whereas Ruch *et al*. have an ET that is organized by the cardiac surgical service.^74^ Regardless of these variations, the ET should convene regularly; weekly meetings seem the appropriate frequency for most centres. However, decisions regarding urgent or unstable patients should not be deferred until the next scheduled meeting but be addressed immediately. The ET should be capable of holding *ad hoc* meetings to accommodate the potential rapidly deteriorating nature of IE cases. Additionally, the complex nature of IE may limit the number of patients that can be discussed per meeting, and more than a weekly meeting might be required in very high-volume centres. Published data from ET centres suggest that the median annual volume of patients with definite IE referred to ET is about 45–50 (range 24–75) (*Table 1*).^32,33^ One member of the ET should be responsible for summarizing key aspects of the patient’s clinical presentation and documenting the ET’s recommendations in the medical record. This note will assist the treating team and other contributors involved in the patient’s care, including the ET itself, if the patient is revisited.

### Implementation

Initiation of the ET requires a group of motivated physicians who are invested in IE, within a supportive institution. Case-finding and self-advertising through direct communication with attending physicians in primary care, IDs, or cardiology may be necessary in the early phases, and requires a devoted ET member. The organizational role may be transitioned to a clinical nurse specialist (CNS) once the ET has become established. Quality improvement metrics, including objective and subjective survey data, generated by the ET are invaluable to the institution.^90^ It is important to keep in mind that mortality is often not impacted in the short-term, and therefore the lack of demonstration of significant benefit in the early phase should not be discouraging. Dissemination of initial quality improvement data, which can include electronic and paper advertisements and word-of-mouth among specialists may help to solidify the role of the ET within the institution. Once the ET is well-established, its goal should be to develop itself into a regional referral centre for IE. The ET is typically based in tertiary centres with on-site cardiac surgery capabilities, but its services should extend beyond these hubs to support referring centres, such as local or community hospitals and remote communities. The regional referral centre not only provides timely access to diagnostic imaging and surgical therapy but also must take advantage of higher IE volumes to foster specialized expertise in cardiac imaging interpretation and complex surgical reconstruction in IE. The COVID-19 pandemic has significantly accelerated the adoption of digital solutions, enabling seamless connectivity among healthcare professionals and facilitating the sharing of imaging and other critical data. The ET’s role includes guiding referring treating teams in selecting the appropriate diagnostic tests and treatments while co-ordinating early transfers when necessary.

### Continuous quality improvement and research

Data collected through the ET can be readily maintained in a longitudinal institutional database and included in large international registries. Continuous review of local data is important to inform regional and institutional practice. Continuous review of quality data also ensures that local practices align with institutional and national guidelines and can improve outcomes when combined with an ET that can quickly adapt to feedback from these data.^70^ The ET should facilitate rapid access to echocardiography in the region. The ET also serves as a foundation for clinical trials that require multi-centre support.

### Patient-centred initiatives and education

Registry data indicates that recurrent IE comprises around 8%–9% of all IE, and that most of the risk of recurrence or relapse is accrued within the first year.^15,16^ Therefore, prevention and early recognition of IE is paramount in reducing the risk of recurrent IE, especially within the first year of the index episode. The ET should strive for a patient-centred approach and assist in improving patient education on all aspects of IE. Adherence to both oral hygiene and antibiotic prophylaxis guidelines are low, as are often adherence to other health maintenance practices.^91,92^ Patients who inject drugs should be made aware of risk reduction practices, including use of sterile injecting equipment, opioid agonist treatment, and drug consumption rooms although this obviously largely beyond the scope of the ET. Education through resources such as pamphlets and reminder cards can empower patients to discuss these topics with their health providers. Patients with IE may be encouraged to serve as awareness advocates and participate in groups to address psychosocial stress related to this diagnosis that are often overlooked.^93^

## Specialty-specific rationale and roles

The expansion of ET reflects the increasingly complex nature of IE. While core group members include a cardiologist, an IDs specialist, and a cardiac surgeon, expert opinion is often required from microbiologists, radiologists, nuclear medicine, neurology, intensivists, anaesthesiologists, AM specialists, electrophysiologists, adult congenital heart disease specialists, advanced nursing specialists, social workers, pharmacists, and primary care providers (*Graphical Abstract*). It must be emphasized that there is no one-size-fits-all approach; flexibility in ET composition should be encouraged. The published experience in ET is biased towards institutions in high- and middle-income countries and does not necessarily reflect the reality of health care provision in lower income countries. There is also an important institution-specific variability; often, the ET must integrate into an existing care model. Although the following section highlights the importance of each main group, it must be acknowledged that other specialists not listed here may contribute to the importance and functioning of the ET, and each individual ET must be tailored to locally available expertise and resources.

### Cardiologist

Cardiologists provide expertise in the recognition of IE and management of resultant valvular disease and heart failure. Expertise in echocardiography among at least one cardiologist member of the team is critical. Offering prompt and precise echocardiography, including TOE, is instrumental in recognition of IE and its complications. Whereas, accurate recognition of false positive artefacts and IE mimics is critical, limitations specific to the echocardiogram for each patient are equally important. For example, prosthetic valves in the aortic position may obscure visualization of the anterior aortic root on TOE, and paravalvular complications in this location may only be identified on a high-quality TTE or CT. Valve disease, ventricular dysfunction, and intracardiac shunts through fistulae secondary to IE are best identified and managed by a cardiologist. The increasing use of transcatheter valve interventions has also increased the incidence of associated IE.^94^ In situations where open surgery is contraindicated or considered at high/prohibitive risk, the use and careful timing of percutaneous devices such as transcatheter aortic valve replacement, edge-to-edge repair, or paravalvular leak closure may be considered once antibiotics course is terminated and there is no sign of active infection potentially in combination with long-term oral suppressive therapy.

### Infectious diseases specialist

The ID specialist provides expertise in both diagnostic and therapeutic aspects of IE management. Common clinical scenarios include non-infectious IE, culture-negative IE, extra-cardiac foci of infection, and identification or unusual or atypical microorganisms that must be distinguished from contamination isolated from blood or other specimen sources. The ID specialist has a crucial understanding of the clinical probability of IE, and must indicate when IE remains strongly suspected despite absence of imaging evidence to suggest infection. The ID specialist also guides appropriate investigation to identify the source of infection. The decision on duration of anti-microbial therapy, need and timing of synergistic combination antibiotic therapy, and mitigating the risk of adverse drug events require timely understanding, especially when surgical therapy or device extraction is planned. The ID specialist plays also a key role in adapting antibiotic doses in cases of renal insufficiency, in the choice of antibiotics in cases of allergy, in evaluating whether a course of antibiotics could be completed at home (outpatient parenteral antibiotic treatment) and the appropriateness of transitioning from intravenous to oral antibiotics in select patients.^40,95^ In patients with IE episodes managed without surgery despite indications for such, decisions regarding whether long-term suppressive therapy is an option and, if so, what drug(s) are appropriate are better made by ID specialists.

### Cardiac surgeon

The cardiac surgeon, the third core member of the ET, should be an experienced specialist with a particular focus and interest in IE. As experts in mitral or aortic valve repair surgery, surgeons dedicated to IE bring critical added value to the surgical treatment of this condition. Working closely with the cardiologist and ID specialists, the cardiac surgeon plays a crucial role in determining the need and optimal timing of surgery. Evidence supports early surgical intervention, and the IE surgeon should align with this approach.^39,52,96,97^ If surgery is indicated, it should not be delayed, especially when there is a risk of severe complications. Mitral valve repair may be preferable to valve replacement but is oftentimes not possible in the acute phase. Most IE cases involve high surgical risk due to the disease’s intrinsic severity, the extent of anatomical damage, associated complications, and the urgency of the intervention. Although the final decision to operate rests with the cardiac surgeon, these decisions should be shared and supported by the ET based on mutual trust between all team members. In particular, the ET informed by the cardiac surgeon should recognize when surgery is futile, and a non-operative or even palliative approach should be considered instead. Intra-operative tissue cultures are essential in guiding duration of anti-microbial treatment. The surgeon should communicate intra-operative findings that were missed by diagnostic imaging or findings that may necessitate adjustments in antibiotic therapy. The surgeon is also responsible for clinical monitoring in the post-op phase to recognize signs of relapse or decompensation, which should be immediately fed back to the ET.

### Electrophysiologist

The growing incidence of CIED infection reflects the increasing number of CIED implants in an aging population and requires input from an electrophysiologist who ideally has expertise in device extraction. Evidence supports prompt CIED removal once an indication is found, as the likelihood of sterilizing CIED lead with antibiotic therapy is low.^98,99^ Although device extraction is associated with reduced mortality, its use remains low.^100^ Safe and successful lead extraction, with or without debulking with percutaneous vacuum devices, requires training and expertise.^61,101^ The electrophysiologist will evaluate the indication for the CIED, assess the risks associated with device extraction, determine the necessity and optimal timing for re-implantation and decide on the most suitable type of device replacement. The indication for and selection of temporary device to bridge prior to definitive re-implantation is based on the patient’s risk of recurrent arrhythmia and pacemaker dependence and weighed against the risk of relapsed infection. Alternate strategies such as implantation of epicardial leads or leadless devices may be considered depending on whether concomitant surgery is indicated.^102,103^ Active collaboration between electrophysiology and ID specialists is essential in complex cases, such as bloodstream infections without detectable vegetations on the CIED leads. These discussions weigh the increased mortality associated with retaining an infected device against the potential complications of unnecessary lead removal. Key factors to consider include the infecting microorganism, the duration of bacteremia, and the challenges involved in lead extraction and re-implantation. Chronic antibiotic suppression is recommended in patients with definite IE who are unsuitable for device extraction. Finally, the electrophysiologist has an important role to play in prevention of CIED infections by making appropriate device selection based on predicted infection risk^104^ and optimizing procedural hemostasis.^105^

### Neurologist

Neurologic complications occur in about 20%–30% of IE, and the majority of these are ischaemic embolic strokes.^57,106,107^ The timing of surgery for patients with severe central nervous system complications can be complex and relies on limited evidence. The neurologist provides expertise in patients who have suffered strokes and have residual vegetations or other indications for surgery. The stroke neurologist should offer a guide to prognosis and likelihood of haemorrhagic transformation of an existing infectious embolus, as it pertains to any competing need for anti-coagulation. Most recent IE guidelines recommend proceeding with surgical therapy in patients with indications for surgery and non-haemorrhagic stroke in the absence of significant severely altered level of consciousness or major cerebral oedema.^5^ Intracranial mycotic aneurysms, present in 1%–9% of IE,^108–110^ have important implications when cardiopulmonary bypass or long-term anti-coagulation is required. Endovascular or open repair should be performed in cases of ruptured mycotic aneurysms or unruptured aneurysms that do not respond to antibiotic therapy.^111^ The optimal management of incidental mycotic aneurysms is not well established. Early data also suggests that mechanical thrombectomy appears to be a feasible therapeutic option for large vessel cerebral occlusion caused by emboli in IE.^112^ Long-term morbidity following stroke is common in IE and requires follow-up through the stroke rehabilitation process.

### Addiction medicine specialist

AM specialists have become invaluable in the era of the growing opioid and IDU epidemic and resultant IDU-associated IE cases. Usual medical or surgical treatment of IDU-associated IE is ineffective without addressing underlying addictions, ongoing IDU, psychological and social issues including food stability, healthcare coverage, personal safety, and housing. Although short-term outcomes are more favourable in patients with IDU-IE than in non-IDU-related IE (younger patients with few comorbid conditions), the IDU-IE patients have a much higher rate of IE recurrence and worse long-term outcomes including death.^113–115^ The AM specialist assists in the assessment, diagnosis, and medical stabilization of SUD, including opioid use disorder (OUD), substance withdrawal, and pain management. The AM specialist also helps guide inform decision-making pertaining to appropriateness and readiness for surgical valve repair or replacement, which is critical when dealing with a relapse or recurrence of IE in younger adults presenting a surgical indication.^116,117^ The willingness to perform surgery in IDU-IE is widely variable across jurisdictions with substantial variation in surgical opinion as to whether surgery should be offered in these patients. Management of OUD in hospital includes both effective opioid agonist therapy as well as longitudinal counselling support, which reduce the incidence, recurrence, and mortality associated with IDU-related bacterial infections, including IE.^13,118,119^ Social services, including housing support and harm reduction programming, as well as longitudinal follow-up with a community-based AM specialist should be instituted prior to discharge to minimize the risk of IDU or IE relapse.^120^

### Clinical nurse specialist

While not part of every ET, a CNS, when available, can be an invaluable asset. By managing logistical tasks, ensuring seamless co-ordination, a CNS significantly enhances the efficiency of the ET and potentially improves outcomes.^121^ The CNS recognize the appropriate attendees for each meeting and carefully considering the additional individuals whose expertise and input are essential. The CNS also facilitates and prioritizes necessary tests to streamline the process and optimize patient care. Communication with providers is a central requirement and the CNS works with the treating team to ensure that the wishes of the patient are respected and those therapies align with the goals of care of the patient. The CNS can also ensure that patients are well-informed about IE providing the necessary education to understand their condition and the available treatment options.

## Challenges and potential solutions

A successful ET requires long-term dedication among members who are both active in patient care and are interested in clinical research-related contributions. Local health care administrators must be made aware of the contributions of the ET to support funding and administrative involvement that are crucial in maintaining the academic, clinical, and organizational needs of the team. Remuneration for participation might facilitate attendance, particularly as multi-disciplinary discussions are now guideline-endorsed. Clarifying inclusion and exclusion referral criteria may help restrict discussion to the most necessary cases such that meeting times are kept to a reasonable timeline but also prevent unnecessary diagnostic or treatment delay in unequivocal cases. Prompt communication of discussion points, key recommendations, and supporting rationale must be provided to the treating team. However, recommendations should acknowledge some flexibility to adapt if the values of the patient change. Local medicolegal agencies may offer valuable insight into how recommendations may be best communicated. As the number of institutions with ET grows, so does the incentive for dedicated cardiovascular infection experts and opportunities for formal training programmes.^122^

## Conclusions

Infective endocarditis is a life-threatening infection characterized by both cardiac and extra-cardiac complications. The diagnosis, investigation and management of IE is increasingly complex and requires expertise in numerous disparate fields. Evidence suggests that ET may improve IE outcomes. Whereas many models of ET exist, flexibility to adapt to institutional and regional practices is paramount. The formation of an ET provides opportunities for mutual learning at a time when cardiovascular care is increasingly specialized and segregated and a framework for research and patient education in an area where these are often lacking.

## Declarations

### Data Availability

Upon request to the corresponding authors.
